# Practical considerations in improving research through public involvement

**DOI:** 10.1186/s40900-015-0002-y

**Published:** 2015-06-25

**Authors:** Martin K Jenner, Mark Gilchrist, Gillian C Baker

**Affiliations:** 1 Royal Devon and Exeter NHS Foundation Trust, Exeter, EX2 5DW UK; 2 Peninsula Research Bank (PRB), NIHR Exeter Clinical Research Facility, University of Exeter Medical School, Exeter, EX2 5DW UK

**Keywords:** Public involvement, Lay steering committees, Tissue banks, Experimental medicine

## Abstract

There is huge commitment to public and patient involvement (PPI) in UK clinical research. Despite there being wide agreement to practice PPI and national guidance on the subject, there are few practical examples of how to implement PPI and few published models to demonstrate how it can be achieved consistently and constructively across a broad portfolio of studies. The Peninsula Research Bank demonstrates how good PPI can be effectively integrated into experimental medicine research.

## Background

When it was established in 2006, the National Institute for Health Research (NIHR) made a bold statement that public involvement would be a core principle in the way it worked. It signalled the beginning of a culture change in how UK health research is conducted [1]. Over the past 8 years, there has been significant investment in public and patient involvement (PPI) and a strong commitment to developing the patient involvement agenda [2]. PPI has been reported in a number of specific research studies and clinical trials [3], and there is excellent generic guidance available from INVOLVE (http://www.invo.org.uk) on the principles of involving the public in research. However, in a recent publication by NIHR on PPI, it was reported that individuals expressed difficulty in understanding clearly what is required for public involvement and how to deliver it [4]. There is no consensus on how to evaluate the effectiveness of PPI [4], and reports of successful PPI rarely provide sufficient operational detail to be duplicated.

In this paper, we present a practical approach to bringing academic and patient communities together to support a portfolio of clinical research studies and provide insights from the viewpoint of both patient and researcher.

### How the PRB Steering Committee has brought researchers and lay representatives together to embed PPI in a research facility: Committee Chair Viewpoint

NIHR funds 19 clinical research facilities around the country to support and facilitate experimental medicine research. NIHR Exeter Clinical Research Facility (CRF) has been running a public involvement and engagement programme over the past 5 years. This programme has evolved from a steering committee setup to advise on means to increase participation in a research biobank (Exeter 10000) and to approve access to blood samples and participant data held in the Peninsula Research Bank (PRB). The steering committee originally had two “nominal lay members”. However, it was soon realised that handing lay members the key to the research bank’s resources was an important step in involving them in research, and the group quickly evolved from nominal lay membership to a PPI group.

What has developed is a co-dependent relationship with researchers and lay members working together in an environment of mutual respect and collaboration. As well as seeking lay approval for access to the research bank, researchers actively seek out our lay members to discuss protocols, information sheets and research ideas and to improve dissemination.

No individual is representative of the public or a particular patient group. Anyone can join the steering committee and there is no limit on overall numbers, but only two to six steering committee lay members attend any single meeting on a rolling basis. Our 27 currently active lay members put themselves forward either having already participated in one of the CRF’s research projects or having attended one of the CRF’s outreach events. All members receive the papers for each meeting 10 days in advance by post or email and can send feedback on proposed projects by email, postal letter or telephone, but there is no expectation for any of them to do this. Members of the public from different walks of life participate on a flexible basis according to their degree of availability, particular expertise and interest in a given project.

Papers can include lay descriptions of the proposed research, patient information sheets, consent forms and any other document on which the researcher would like a broader perspective. All comments received by email or telephone are collated and sent to researchers so they can provide answers either before or during the meeting. Minutes detailing all comments and researcher responses are collated and distributed within 10 days of the meeting, and often, access to samples or volunteer data is subject to conditions imposed by the steering committee. We do not choose individuals with specific expertise or personal qualities, have no “Job Description” and do not provide training or make training a requirement of participation. The agenda for each meeting clearly specifies what involvement is required and which items are essential reading. In addition, every meeting has an agenda item entitled “Lay member ideas”, which is an open forum for lay members to steer our PPI activity. If lay members on our steering committee wish to undergo training, we refer them to other organisations, such as PenCLAHRC and the Research Network who provide courses and training days. Our method of lay involvement promotes an interesting balance of representatives, from those with no formal education keen to promote NHS research to their friends and neighbours to retired professionals with the skills to question scientists about experimental design and statistical validity.

Academic culture can often be very different from that of other spheres of work, and bringing lay representatives into the research arena helps to raise awareness of issues outside the academic culture box. This is particularly useful where researchers have moved through first and second degrees into doctorates and post-doctoral research in basic science. Lay members are more focussed on the practical aspects and outcomes of research and how it can affect patients and carers. They raise practical questions about study design, such as during a long study visit when can participants drink or visit the toilet? Taking time to consider these practicalities ensures that study visits run more smoothly. This means that participants feel better cared for and are more likely to attend follow-up visits and participate in further research. The impact that lay members have had on the content and format of participant information sheets has been significant. Suggestions such as “why don’t you include the consent statements in the information sheet, so we know exactly what we are being asked to sign up to” not only provide clarity for potential participants but speed up and improve the process of obtaining informed consent. Lay members are interested in the translational outcomes of research and help researchers to think through how their research can improve healthcare. Close and regular dialogue can help researchers to consider these outcomes and to prepare intelligible lay summaries of their research that will help generate realistic timescales and avoid exaggerated forecasts of impact. This, in turn, may assist in their applications for funding.

A key feature of our model is that all research projects running through our facility are receiving involvement from a wide range of lay members. Individual lay members may get involved at a deeper level in specific projects, but they have broad involvement in everything that we do which means that on a monthly basis, there is always an opportunity for researchers and lay members to share opinions and ideas. This differs from many PPI models where individual projects are allocated one or two lay members whose involvement may go through peaks and troughs.

### Lay member viewpoint from Martin Jenner

There are ever increasing opportunities for public and patient involvement in healthcare research and service delivery. The demand currently outstrips supply, and there are many local and national initiatives whose objectives include, or are solely focused on, correcting this imbalance. One of the difficulties is the proliferation of ideas about what constitutes PPI. As organisations accept the need for lay involvement, or have it thrust upon them, they struggle to grasp the concept or resist the feeling of intrusion. I believe that it is not possible to write a job description that avoids the idea of constricting the contribution of lay participants. Organisations need to adopt an open-minded approach to how the general public through PPI can help them to achieve their objectives; Non-executive Board Members are a valuable resource in most companies. The special expertise that builds up in academic and administrative organisations can inhibit innovative (out-of-the-box) thinking, and lay persons can often bring this to benefit early-stage research design and programme delivery.

There are few limits other than those imposed by bureaucratic thinking. There is nothing more damaging to realising the real potential than “tokenism”. I am pleased that I am able to participate in the NIHR Exeter CRF’s PRB as a lay member. It has a clear vision of how PPI fits into its operation and has evolved a practical, effective implementation. It works and it is enjoyable.

The PPI team (they all have other roles) has generated an environment in which everyone feels able to contribute with the certainty that their views and comments will be valued. The communication channels between lay members and researchers are open on all subjects (there is an absence of “no-go” areas) and are truly bi-directional. By sympathetic chairing of meetings, fast and encouraging feedback and the genuine enthusiasm of the research community in the CRF in Exeter, the PRB PPI programme has overcome the initial pitfalls and has no shortage of lay participation. Above all, they are constantly seeking to improve the experience for researchers and lay persons alike. It really feels like one family group all striving to achieve the same objective: ground-breaking research that is understandable, accessible and relevant to the wider community.

### Researcher viewpoint from Mark Gilchrist

Early in my research career, I had the chastening experience of being unable to recruit a single subject to one arm of a three-arm study. The other two arms were recruited without difficulty. The consistent feedback from the patient group required for the third arm was that on top of their regular visits to hospital; the study visits would be an unacceptable burden. I soon realised that PPI groups are better placed to decide whether what is being asked of them in research participation is reasonable and worthwhile. They will often raise issues that neither the originating investigators nor ethics committees had considered. Subsequently, I have proactively involved potential research participants and/or experienced service users at an early stage in my study design.

It has been educational to me to see the divergent opinions of the patients in PPI groups and ethics committees. One area that has generated considerable debate is patient information sheets. PPI groups have consistently asked for more concise information sheets, whereas some ethics committees prefer longer more detailed information sheets. From discussions with PPI groups, it is evident that a brief, clear information sheet is more likely to be read and understood than a more exhaustive document. It is helpful in ethics meetings to be able to answer questions from the committee with evidence from a PPI group.

Research participants will only take part in studies which they deem to be of value. PPI groups give the researcher the chance to explain their work and its importance to an audience of individuals who are experienced in particular medical conditions. There is scope for learning what will concern potential participants and what may enhance or hinder recruitment and retention.

## Conclusions

The model for PPI adopted by the PRB at NIHR Exeter CRF provides a consistent and constructive framework for lay members and researchers to work together to improve experimental medicine research. This model can be applied to any organisation with a portfolio of several research studies for which broad lay involvement is needed. It is also an ideal spring board to involve lay members more deeply in specific projects.

Key to the operational running of this model is central management playing a facilitative role between researchers and lay representatives. The NIHR Exeter CRF provides less than 1 day a week of staff time to support to manage this relationship and to ensure that the 30–50 studies per year have public and patient involvement. Where an organisation has a full-time member of staff involved in PPI, they should be able to use this model to embed broad PPI in a large portfolio of research and can use this as an opportunity to develop deeper involvement in studies where the public can play a vital role as co-applicants.

Our model is particularly effective to use where lay members have donated samples to a tissue bank or joined a research register, as they are specifically interested in ensuring samples/data are used for the very best research.

There has been debate surrounding the relative merits of broad or dynamic consent for tissue banks [5]. Broad consent asks participants to consent to their samples being used for any purpose within a broad category, such as to be used in “diabetes research”. Dynamic consent involves taking the samples and then going back to individual donors and asking them to re-consent each time their samples are used.

The PRB obtains broad consent from all donors, but all projects using samples or data within the bank are scrutinised by the lay steering committee (PPI group), who are themselves donors. This approach results in a proportion of the participants being engaged in dynamic consent. Ensuring dynamic consent for all donors in a tissue bank is an administratively difficult and costly exercise, as donors change their contact details. Confirming that all participants understand what is involved in every research project would also be operationally difficult in a large biobank, as truly informed consent would involve them being able to ask questions of the researcher, which is not feasible when a researcher wants to use 1000 s of DNA samples. In addition, many donors prefer not to be bombarded with specific requests and paperwork. As membership of the PRB is open to anyone, including any person who has donated samples to the bank, it allows individuals with an interest in what is being done with their samples to have a voice in approving access on behalf of the whole cohort. This model of broad consent plus “dynamic consent by proxy” improves the ethical standard of the biobank but also provides a practical, consistent and constructive framework for involving the public in research, as regular meetings are needed between lay members and scientists to approve access to samples. The PRB started as a committee to approve access to samples, but developed into a PPI group, as lay members started asking to be involved more deeply in the studies that they were approving and researchers realised that early dialogue improved their study design and participant-facing materials.

For more information on the operational details of the PRB Steering Committee, such as copies of minutes and example information sheets, see www.exeter.crf.nihr.ac.uk/PRB.

## Abbreviation

PenCLARHC: Peninsula Collaboration for Leadership in Applied Health Research and Care
